# The International Medical Annual and Practitioners’ Index

**Published:** 1894-05

**Authors:** 


The International Medical Annual and Practitioners' Index: A work of reference for medical practitioners. Twelfth year, 1894. New York: E. B. Treat, 5 Cooper Union. Chicago: 199 Clark Street. Price, $2.75.

Every year this valuable annual, containing the latest discoveries, views, and methods of treatment of the diseases that affect humanity comes out and is brought to the attention of the readers of this journal. The present volume rather exceeds in fullness, copiousness of illustration, and beauty of execution, its predecessors.

It has less the character of a digest, and more the value of an encyclopedia by the number of direct communications it contains from the various authorities whose progressive views and methods are recorded. Indeed, the printing of these original communications is the especial feature of the book. It is finely and profusely illustrated by photographs and colored plates. It is a matter of regret that the reviewer is not able to discuss the contents in order and extensively. But that would take more time than we can hope to bestow. And then it would not be satisfactory. The best way is to purchase the book and see for yourself what it is like. Our word may be taken without misgiving that it is well worthy a place in the library; and this is all that need be said.

Our readers well know that we do not recommend books devoid of merit.

